# Interpreting Cytomegalovirus Seropositivity Beyond the Neonatal Diagnostic Window: A Case Report on an Infant With Early-Onset Atopic Dermatitis and Congenital Arthrogryposis

**DOI:** 10.7759/cureus.116716

**Published:** 2026-09-22

**Authors:** Auda Ameur, Charef Latroch, Karim Bouziane Nedjadi, Amel Khelil, Mohammed Assameddine Adnane

**Affiliations:** 1 Department of Pediatrics B, Amilcar Cabral, University of Oran 1, Oran, DZA; 2 Department of Dermatology, University of Oran 1, Oran, DZA; 3 Radiology and Imaging of Central Nervous System, Tourabi Medical Imaging and Radiodiagnosis Center, Oran, DZA

**Keywords:** algeria, atopic dermatitis (ad), congenital cytomegalovirus, microcephaly, early diagnosis

## Abstract

Congenital cytomegalovirus (cCMV) infection has a broad clinical spectrum and may result in permanent neurological, auditory, visual, and developmental sequelae. We report the case of a six-month-old male infant investigated primarily for microcephaly who had a complex phenotype comprising a cutaneous rash present from birth that evolved into moderate early-onset atopic dermatitis with severe clinical and quality-of-life impairment, growth impairment, congenital arthrogryposis, neonatal jaundice with hyperbilirubinemia, mild neonatal thrombocytopenia, and neurosensory abnormalities. He was born at 36 weeks and four days after reduced amniotic fluid volume was noted at approximately 36 weeks. Neonatal hyperbilirubinemia did not require phototherapy, and a follow-up peripheral blood smear was unremarkable.

At six months, the dermatitis consisted of widespread erythematous-squamous, intensely pruritic lesions with facial and periorbital involvement and sleep disturbance. After 40 days of conventional treatment with emollients, skin-barrier care, and topical corticosteroids, the SCORAD (Scoring Atopic Dermatitis) index decreased from 45 to 18, although residual lesions persisted. Growth was impaired, with marked underweight (weight-for-age z-score <−3) and reduced linear growth (length-for-age z-score −2.6), while head circumference was 40.8 cm (z-score −2.2), consistent with microcephaly. Brain magnetic resonance imaging showed preserved cerebral architecture with symmetric periventricular and peritrigonal T2 hyperintensity considered indeterminate and potentially related to incomplete physiological myelination. Auditory brainstem evoked responses and visual evoked potentials were abnormal. Creatine kinase was mildly elevated, whereas neurological examination and electromyography were normal. Quantitative immunoglobulins, serum protein electrophoresis, and lymphocyte subset analysis were unremarkable.

Maternal CMV-specific IgM and IgG were positive during the eighth month of pregnancy, and the infant had positive CMV-specific IgM and IgG at six months. In the absence of virological testing within the first 21 days of life or an archived neonatal dried blood spot, congenital infection could not be definitively confirmed. This case emphasizes the critical importance of recognizing suggestive neonatal findings and testing for cCMV within the first 21 days of life, particularly in settings where retrospective dried-blood-spot testing is unavailable.

## Introduction

Congenital cytomegalovirus (cCMV) infection is one of the most common congenital viral infections and may result in fetal growth restriction, microcephaly, neurodevelopmental impairment, sensorineural hearing loss, visual abnormalities, hematological abnormalities, and other multisystem manifestations [1-3]. Contemporary European consensus recommendations emphasize accurate prenatal assessment, virological confirmation during the neonatal diagnostic window, and risk-adapted long-term follow-up [1].

Classical cutaneous manifestations of symptomatic cCMV include petechiae, purpura, and, less commonly, a “blueberry muffin” eruption associated with dermal extramedullary hematopoiesis [4,5]. A recent 2026 case report further highlighted blueberry-muffin-like lesions as a rare dermatological presentation of cCMV [5]. In contrast, generalized intensely pruritic eczematous dermatitis is not recognized as a classic CMV-specific cutaneous phenotype and should prompt consideration of a concomitant inflammatory dermatosis, genetic skin-barrier disorder, or inborn error of immunity.

This case highlights the importance of considering congenital infections and performing targeted evaluation for TORCH pathogens (*Toxoplasma gondii*, rubella virus, cytomegalovirus (CMV), and herpes simplex virus (HSV)), Zika virus, and parvovirus B19 when clinically indicated in neonates and young infants with unexplained congenital or neonatal abnormalities. Particular attention should be given to cCMV because the first 21 days of life represent a critical diagnostic window for reliably distinguishing congenital from postnatally acquired CMV infection [1]. Early recognition enables timely assessment of disease severity, consideration of antiviral therapy when indicated, and structured audiological, ophthalmological, and neurodevelopmental surveillance. This diagnostic window is particularly important in healthcare settings where neonatal dried blood spots are not routinely collected or stored, making retrospective virological confirmation difficult or impossible [6]. We report an infant with a rash present from birth that subsequently evolved into severe early-onset atopic dermatitis, associated with microcephaly, congenital arthrogryposis, neonatal jaundice and thrombocytopenia, growth impairment, and neurosensory abnormalities, in whom probable cCMV infection was recognized only at six months of age.

## Case presentation

Prenatal and perinatal history

The patient was a male infant born prematurely at 36 weeks and four days of gestation and first evaluated at six months of chronological age. Reduced amniotic fluid volume was reported at approximately 36 weeks of gestation, and congenital arthrogryposis with limb joint contractures was noted at birth.

Maternal CMV serology obtained during the eighth month of pregnancy showed positive CMV-specific IgM and IgG antibodies. However, preconception serological results, documented IgG seroconversion, CMV IgG avidity testing, and fetal CMV PCR were unavailable. Consequently, neither the timing nor the primary nature of the maternal infection could be established. This serological pattern could represent primary infection, CMV reactivation or reinfection in a previously seropositive mother, persistent IgM following an earlier infection, or a nonspecific IgM result.

A cutaneous rash was present at birth. The neonatal course was also notable for jaundice associated with hyperbilirubinemia (total bilirubin, 23 mg/L), which did not require phototherapy, and mild thrombocytopenia (platelet count, 120 × 10⁹/L). A follow-up peripheral blood smear showed no morphological abnormalities.

Neither CMV PCR nor viral culture was performed during the first 21 days of life. The etiological work-up was initiated at six months, primarily because of microcephaly. An archived neonatal dried blood spot was unavailable for retrospective CMV testing.

The infant was breastfed until six months of age, after which mixed feeding and age-appropriate complementary feeding were introduced. No clinically apparent food allergy was observed following dietary diversification.

Dermatological presentation

At the initial evaluation at six months of age, dermatological examination revealed widespread erythematous-squamous eczematous lesions involving the face, including the periorbital region, neck, trunk, and extremities. The eruption was intensely pruritic and associated with incessant crying and marked sleep disturbance. Based on the morphology and distribution of the lesions, a clinical diagnosis of early-onset atopic dermatitis was made (Figure 1).

**Figure 1:**
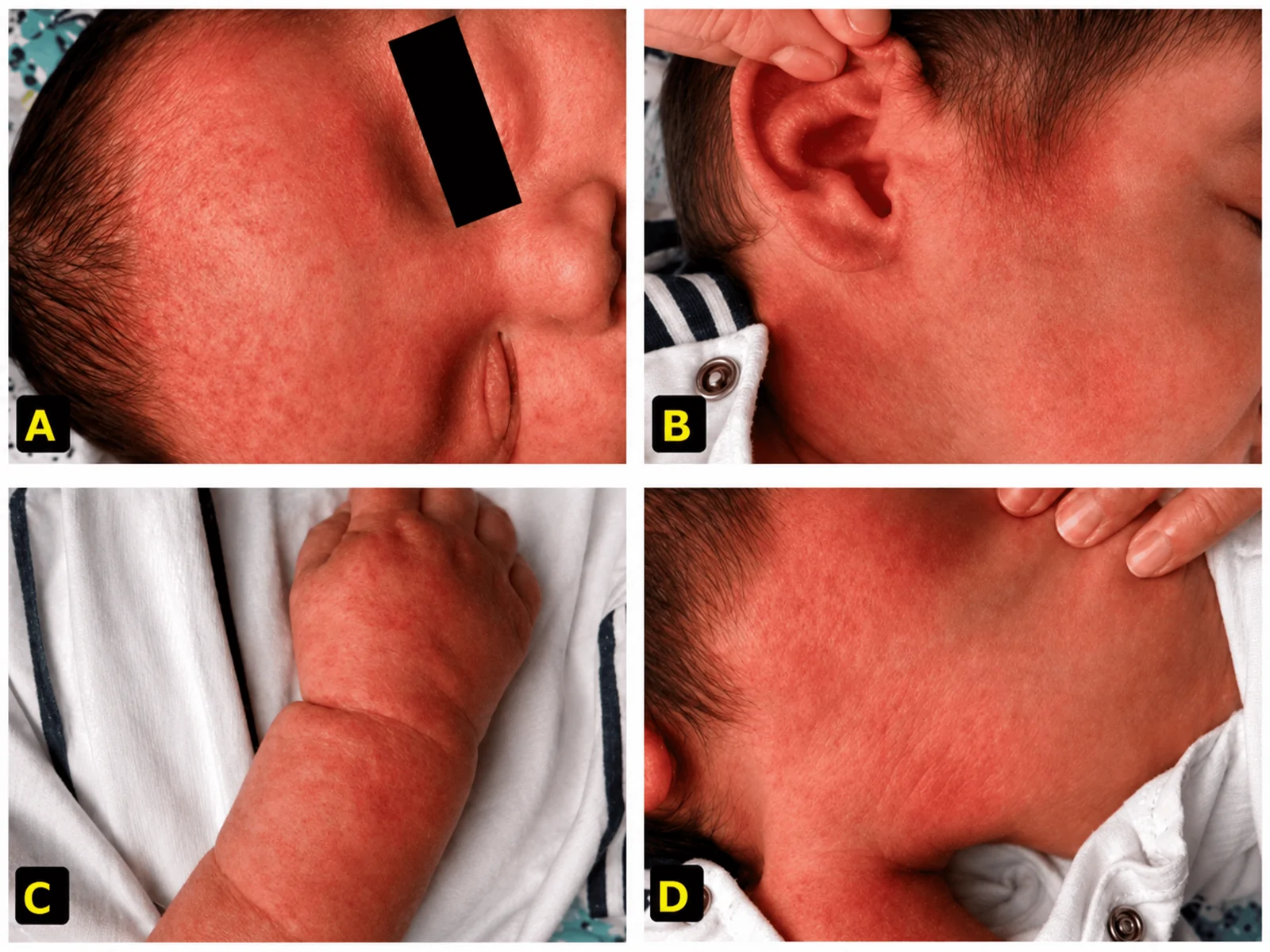
Atopic dermatitis during early infancy. (A) Erythematous and eczematous lesions involving the cheeks. (B) Erythema and eczematous lesions of the auricular and retroauricular areas. (C) Erythematous and scaly lesions on the extensor surfaces of the upper limb. (D) Eczematous lesions involving the neck.

The family history was notable for atopic disease: the patient’s older sister had physician-diagnosed asthma requiring long-term controller therapy, and his father had seasonal allergic rhinitis.

The baseline SCORAD (Scoring Atopic Dermatitis) index [7] was 45, corresponding to moderate atopic dermatitis according to the conventional score-based classification. Nevertheless, the condition had a severe clinical and quality-of-life impact, with widespread intensely pruritic lesions, incessant crying, marked sleep disturbance, and a substantial burden on both the infant and his family.

Treatment initiated at the first evaluation consisted of regular emollient therapy, skin-barrier care, and topical corticosteroids during inflammatory flares. After 40 days, the SCORAD score decreased to 18, corresponding to mild disease and representing a 60% reduction from baseline. Clinical improvement was substantial but incomplete, with residual eczematous lesions. This 40-day treatment period occurred within the subsequent six-month follow-up. There was no history of chronic diarrhea or recurrent infections.

Because of the congenital onset and substantial clinical burden of the dermatitis, together with the associated poor growth, alternative diagnoses were considered. Netherton syndrome was considered because it may present with congenital erythroderma, failure to thrive, and atopic manifestations. However, characteristic ichthyosis linearis circumflexa and clinically apparent hair abnormalities were not identified. Hair-shaft microscopy for trichorrhexis invaginata and SPINK5 genetic testing were not performed; therefore, Netherton syndrome could not be formally excluded.

Infantile seborrhoeic dermatitis was considered less likely because of the extensive, intensely pruritic eczematous involvement and clinical response to emollients and topical corticosteroids. Scabies was considered clinically unlikely in the absence of characteristic burrows, typical acral involvement, or affected household contacts; however, skin scraping was not performed. Zinc or biotin deficiency was considered less likely because there was no alopecia, persistent diarrhea, or typical periorificial or acral dermatitis. Serum zinc and biotin levels and skin biopsy were not obtained. The absolute eosinophil count was also unavailable. Consequently, these alternative causes of early-onset dermatitis could not all be formally excluded.

Growth, neurological, and neurosensory assessment

At six months of chronological age, the infant weighed 4.7 kg, corresponding to a weight-for-age z-score below −3, and measured 62 cm in length, corresponding to a length-for-age z-score of approximately −2.6. These findings indicated marked underweight and impaired linear growth. His head circumference was 40.8 cm, corresponding to a z-score of approximately −2.2 for age and sex and consistent with microcephaly.

Because the infant had been born at 36 weeks and four days of gestation, his corrected age was approximately five months; however, chronological age was used consistently throughout the report.

There was no clinical or radiological evidence of craniosynostosis or intracranial hypertension. Neurological examination was normal. Serum creatine kinase was mildly elevated at 329 U/L, although the original laboratory reference range was unavailable for review. Electromyography was normal, with no evidence of an overt peripheral neuromuscular disorder.

Brain magnetic resonance imaging (MRI) performed at six months showed preserved cerebral architecture without a major structural abnormality, ventriculomegaly, mass effect, or abnormality of the midline structures. Symmetric periventricular and peritrigonal T2 hyperintensity was observed. Given the patient’s age, this appearance may correspond to normally unmyelinated terminal zones or incomplete physiological myelination rather than a pathological white-matter lesion. FLAIR (fluid-attenuated inversion recovery) imaging was considered to have limited discriminatory value at this developmental stage. The MRI appearance was therefore regarded as indeterminate and was not considered evidence of CMV-related white-matter injury (Figure 2).

**Figure 2:**
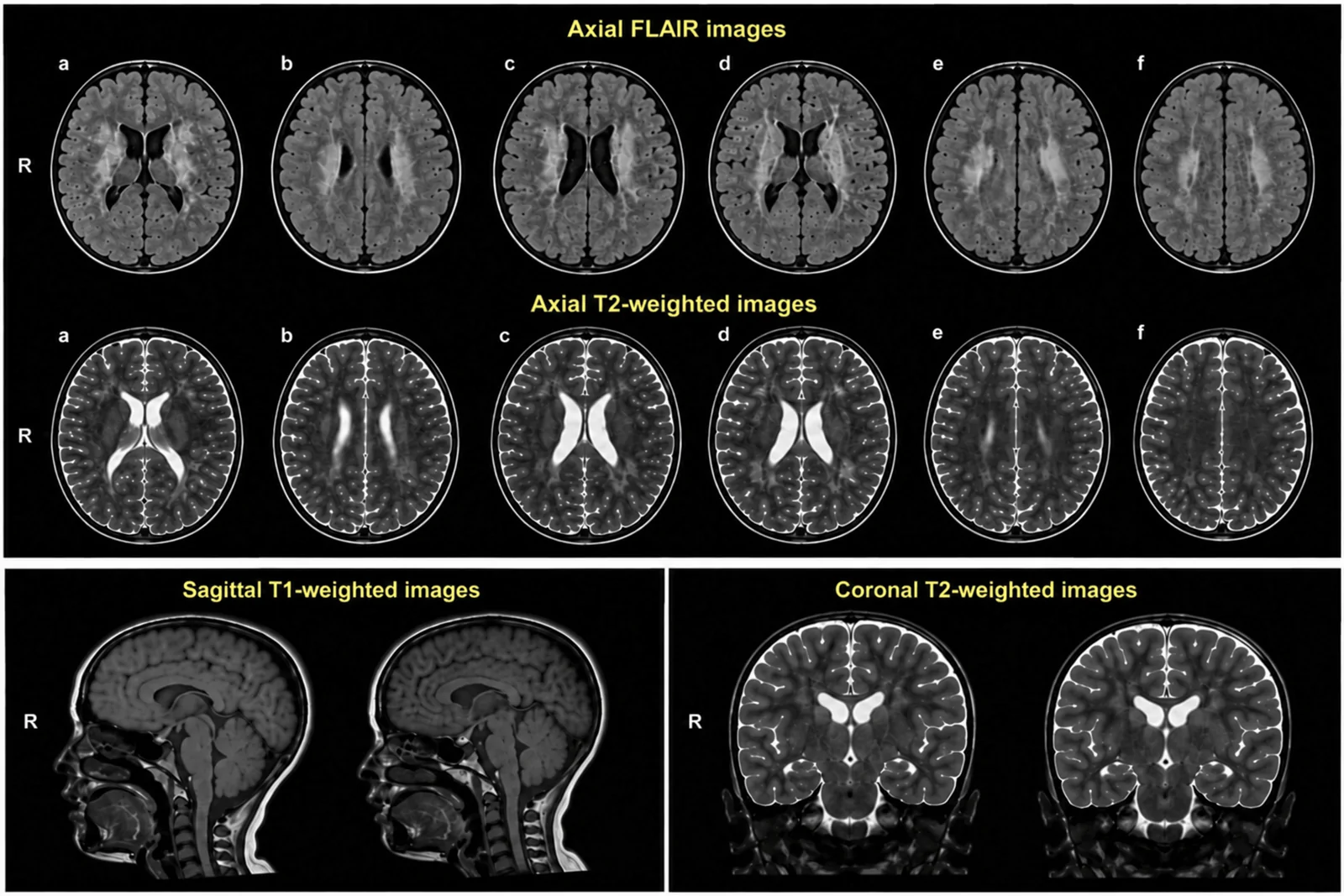
Brain magnetic resonance imaging at six months of age. Multisequence brain MRI demonstrates preserved cerebral architecture without ventriculomegaly, mass effect, or a major structural abnormality. Axial T2-weighted images show symmetric periventricular and peritrigonal hyperintensity, which may reflect normally unmyelinated terminal zones or incomplete physiological myelination at this age rather than pathological white-matter injury. FLAIR imaging has limited discriminatory value at this developmental stage. Sagittal T1-weighted images demonstrate preserved midline structures and normal morphology of the corpus callosum. R indicates the right side. MRI, magnetic resonance imaging; FLAIR, fluid-attenuated inversion recovery.

Auditory brainstem response testing was performed using monaural rarefaction-click stimulation at increasing intensities ranging from 70 to 110 dB HL. Bilaterally, the recordings were poorly reproducible and showed markedly disorganized or attenuated waveforms, making the cochlear and subcollicular generators difficult to identify. Prolonged absolute latencies and increased I-III, III-V, and I-V interpeak latencies were reported. The neurophysiological interpretation suggested bilateral involvement of the peripheral and central bulbopontine auditory pathways, predominantly affecting central conduction and more pronounced on the right. Repeat auditory brainstem response testing after six months was recommended.

Visual evoked potentials showed delayed conduction suggestive of visual pathway dysfunction. Ophthalmological examination, including dilated fundoscopy, was unremarkable, with no evidence of chorioretinitis or clinically apparent optic atrophy.

Continued neurological, neurodevelopmental, audiological, and ophthalmological surveillance was planned.

Skeletal assessment

A skeletal survey demonstrated diffusely reduced bone mineralization with a gracile appearance of the long bones. Multiple limb contractures were evident, consistent with congenital arthrogryposis. No focal destructive osseous lesion or acute fracture was identified (Figure 3).

**Figure 3:**
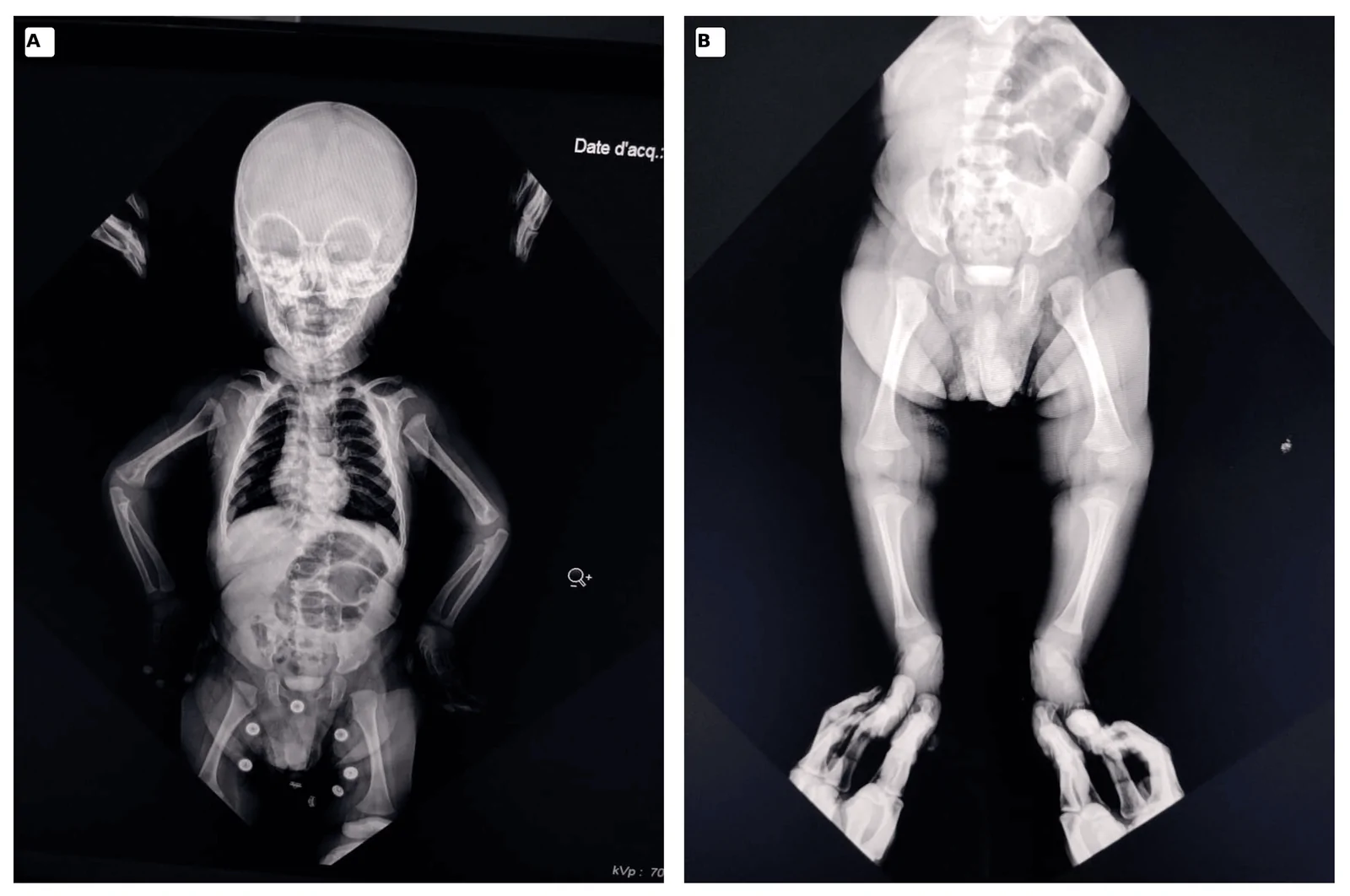
Skeletal survey showing reduced bone mineralization and limb contractures. (A) Anteroposterior whole-body radiograph showing a gracile appearance of the long bones and abnormal positioning of the upper and lower limbs. (B) Dedicated lower-limb radiograph showing bilateral positioning abnormalities and multiple joint contractures consistent with congenital arthrogryposis. No focal destructive osseous lesion or acute fracture is identified.

Serum vitamin D levels and calcium-phosphate parameters were within the age-appropriate reference ranges, providing no biochemical evidence of vitamin D deficiency or a calcium-phosphate metabolism disorder accounting for the radiographic findings.

Comprehensive genetic investigations, including an arthrogryposis or neuromuscular gene panel and exome sequencing, were not performed. Therefore, an underlying congenital neuromuscular or syndromic disorder could not be formally excluded.

Immunological, metabolic, and infectious work-up

Given the unusually early onset and substantial clinical burden of the dermatitis associated with poor growth, an underlying immune disorder was investigated. Quantitative serum immunoglobulin levels were within age-appropriate reference ranges (IgG, 4.8 g/L; IgA, 0.28 g/L; IgM, 0.72 g/L; and total IgE, 10 kU/L). Serum protein electrophoresis was unremarkable, and lymphocyte subset phenotyping showed no significant abnormalities (CD3⁺ T cells, 3,500 cells/µL; CD4⁺ T cells, 2,300 cells/µL; CD8⁺ T cells, 1,050 cells/µL; CD19⁺ B cells, 1,200 cells/µL; and CD16⁺/CD56⁺ NK cells, 350 cells/µL). Liver, renal, and thyroid function tests were within age-appropriate reference ranges. Serum 25-hydroxyvitamin D (37 ng/mL) and total serum calcium (87 mg/L) were also within age-appropriate reference ranges.

At six months, serum CMV-specific IgM and IgG antibodies were positive. Serological investigations for toxoplasmosis, rubella, herpes simplex virus, Zika virus, and parvovirus B19 were negative.

Urine, saliva, and quantitative blood CMV PCR were not performed at six months. Repeat CMV IgG testing after 12 months was also unavailable. Therefore, CMV infection remained unconfirmed, and the available findings could not reliably distinguish congenital from postnatally acquired infection.

The infant’s principal anthropometric, laboratory, serological, ophthalmological, and neurophysiological findings are summarized in Table 1.

**Table 1:** Summary of the infant’s anthropometric, laboratory, serological, and neurophysiological findings. Anthropometric z-scores were calculated according to the World Health Organization growth standards using the infant’s chronological age of six months. Laboratory results should be verified against the original assay-specific and age-appropriate reference ranges. ABR, auditory brainstem response; CMV, cytomegalovirus; HSV, herpes simplex virus; Ig, immunoglobulin; MRI, magnetic resonance imaging; NK, natural killer; WHO, World Health Organization.

Domain	Timing	Parameter	Result
Anthropometry	At 6 months	Weight	4.7 kg; WHO weight-for-age z-score <−3
	At 6 months	Length	62 cm; WHO length-for-age z-score −2.6
	At 6 months	Head circumference	40.8 cm; WHO z-score −2.2
Neonatal laboratory findings	Neonatal period	Total bilirubin	23 mg/L
	Neonatal period	Platelet count	120 × 10⁹/L
Neuromuscular assessment	At 6 months	Serum creatine kinase	329 U/L
	At 6 months	Electromyography	Normal
Immunological assessment	At 6 months	IgG	4.8 g/L
	At 6 months	IgA	0.28 g/L
	At 6 months	IgM	0.72 g/L
	At 6 months	Total serum IgE	10 kU/L
	At 6 months	CD3⁺ T cells	3,500 cells/µL
	At 6 months	CD4⁺ T cells	2,300 cells/µL
	At 6 months	CD8⁺ T cells	1,050 cells/µL
	At 6 months	CD19⁺ B cells	1,200 cells/µL
	At 6 months	CD16⁺/CD56⁺ NK cells	350 cells/µL
Nutritional and metabolic assessment	At 6 months	Total serum calcium	87 mg/L
	At 6 months	Serum 25-hydroxyvitamin D	37 ng/mL
	At 6 months	Serum ferritin	53 ng/mL
CMV serology	At 6 months	CMV IgM	6.16; positive
	At 6 months	CMV IgG	42 AU/mL; positive
Other infectious serology	At 6 months	Toxoplasma IgM	0.26; negative
	At 6 months	Toxoplasma IgG	<0.130 IU/mL; negative
	At 6 months	Rubella IgG	0.03; negative
	At 6 months	Rubella IgM	0.38; negative
	At 6 months	HSV-1 IgG	0.236 E/S; nonreactive
	At 6 months	HSV-2 IgG	0.095 E/S; nonreactive
	At 6 months	HSV-1/2 IgM	0.06 U; nonreactive
	At 6 months	Zika virus IgG	Index 0.06; negative
	At 6 months	Zika virus IgM	Index 0.04; negative
	At 6 months	Parvovirus B19 IgG	Index 0.54; negative
	At 6 months	Parvovirus B19 IgM	Index 0.86; negative
Neuroimaging	At 6 months	Brain MRI	Preserved cerebral architecture; symmetric periventricular and peritrigonal T2 hyperintensity
Audiological assessment	At 6 months	ABR stimulus intensity	70-110 dB HL; monaural rarefaction-click stimulation
	At 6 months	Right ear	Poorly reproducible, disorganized, and attenuated waveforms; threshold not established
	At 6 months	Left ear	Poorly reproducible, disorganized, and attenuated waveforms; threshold not established
	At 6 months	Interpeak latencies	Increased I-III, III-V, and I-V intervals bilaterally
Visual assessment	At 6 months	Visual evoked potentials	Delayed conduction
	At 6 months	Dilated fundoscopy	Unremarkable; no chorioretinitis or clinically apparent optic atrophy

Clinical follow-up

The infant was followed for six months after the initial evaluation, from six to approximately 12 months of chronological age. The 40-day dermatological treatment course occurred within this follow-up period. Following optimization of nutritional support, weight gain improved. In contrast, microcephaly persisted, with stagnation of head circumference growth during follow-up. No clinical signs of intracranial hypertension were observed.

Given the persistent microcephaly, lack of expected head-circumference progression, and neurophysiological abnormalities, close longitudinal monitoring of head growth, neurological status, neurodevelopmental milestones, hearing, vision, joint contractures, and dermatitis was maintained. Repeat auditory brainstem response testing was recommended approximately six months after the initial neurophysiological assessment.

## Discussion

This case illustrates the diagnostic challenges associated with a complex multisystem phenotype in an infant whose CMV infection was investigated only after the neonatal diagnostic window. Microcephaly, impaired growth, neonatal jaundice, thrombocytopenia, hearing impairment, and central nervous system abnormalities are recognized manifestations of symptomatic cCMV infection [1-3]. Although the coexistence of several findings from birth or early infancy may raise clinical suspicion, none is sufficiently specific to replace appropriately timed virological confirmation.

Maternal CMV-specific IgM and IgG were positive during the eighth month of pregnancy. However, without preconception serology, documented IgG seroconversion, or CMV IgG avidity testing, neither the timing nor the primary nature of the maternal infection could be established. This serological pattern could reflect primary infection, reactivation, or reinfection in a previously seropositive mother, persistent IgM following an earlier infection, or a nonspecific IgM result. Maternal serology therefore provided evidence of CMV exposure but could not confirm primary infection during pregnancy.

Similarly, positive CMV-specific IgM and IgG in the infant at six months could not distinguish congenital from postnatally acquired infection. This distinction is particularly important because the infant was breastfed until six months of age, after which mixed feeding and age-appropriate complementary feeding were introduced. Breast milk from a seropositive mother represents a common route of postnatal CMV transmission and therefore provides a plausible alternative explanation for the infant’s positive serology at six months. However, the cutaneous rash and arthrogryposis were documented at birth and thus preceded any possible postnatal acquisition through breastfeeding.

The absence of CMV PCR or viral culture during the first 21 days of life and the unavailability of an archived neonatal dried blood spot prevented confirmation of congenital infection [1,6]. Nevertheless, the loss of the neonatal diagnostic window did not eliminate all subsequent diagnostic options. At six months, urine or saliva CMV PCR could have assessed ongoing viral shedding, while quantitative blood CMV PCR could have supported active infection. A positive result after three weeks of age, however, would not have distinguished congenital from postnatally acquired infection. Repeat CMV IgG testing after 12 months could also have helped distinguish persistent antibody production by the infant from passively acquired maternal antibodies. These investigations were not performed. Consequently, congenital CMV infection remained unconfirmed.

The present case emphasizes the consequences of missing the early diagnostic window. The infant had a rash and arthrogryposis at birth, neonatal jaundice with hyperbilirubinemia, mild thrombocytopenia, and subsequently recognized microcephaly and impaired growth, yet CMV testing was not performed during the first 21 days. A low threshold for targeted neonatal CMV testing should therefore be considered in newborns presenting with otherwise unexplained petechiae or rash, jaundice, thrombocytopenia, microcephaly, growth restriction, hepatosplenomegaly, or neurological or sensory abnormalities. This is particularly important in settings where systematic neonatal CMV screening and retrospective testing of archived dried blood spots are not routinely available. Early diagnosis permits virological confirmation, appropriate disease staging, timely assessment of eligibility for antiviral treatment, and structured audiological, ophthalmological, and neurodevelopmental surveillance.

The neuroimaging findings require age-appropriate interpretation. Brain MRI performed at six months showed preserved cerebral architecture without a major structural abnormality. The symmetric periventricular and peritrigonal T2 hyperintensity may correspond to normally unmyelinated terminal zones or incomplete physiological myelination rather than pathological white-matter injury. FLAIR imaging also has limited discriminatory value at this developmental stage. Although white-matter abnormalities are recognized within the neuroimaging spectrum of cCMV [8,9], the findings in this infant were considered indeterminate and should not be interpreted as evidence of CMV-related central nervous system injury.

Despite the absence of a major structural brain abnormality, neurophysiological testing demonstrated functional abnormalities. Auditory brainstem responses showed poorly reproducible, disorganized, and attenuated waveforms bilaterally, with prolonged absolute latencies and increased I-III, III-V, and I-V interpeak latencies. The neurophysiological interpretation suggested bilateral involvement of the peripheral and central bulbopontine auditory pathways, predominantly affecting central conduction and more pronounced on the right. Visual evoked potentials showed delayed conduction suggestive of visual pathway dysfunction. Nevertheless, dilated fundoscopy was unremarkable, with no evidence of chorioretinitis or clinically apparent optic atrophy. These findings are not necessarily discordant because visual evoked potentials assess functional conduction along the visual pathways, whereas fundoscopy evaluates visible structural abnormalities of the retina and optic disc. Neither the MRI appearance nor the neurophysiological abnormalities are specific for CMV, and they should not be considered confirmatory evidence of congenital infection.

The dermatological phenotype should be considered separately. Classical cutaneous manifestations of symptomatic cCMV are predominantly petechial, purpuric, or “blueberry muffin”-like [4,5]. Although a rash was present at birth, the dermatitis documented at six months was predominantly eczematous, widespread, intensely pruritic, and responsive to conventional anti-inflammatory skin treatment. The previously documented family history of physician-diagnosed asthma in the patient’s sister and seasonal allergic rhinitis in his father provided additional support for an atopic predisposition.

The baseline SCORAD score was 45, corresponding to moderate atopic dermatitis according to the conventional score-based classification. Nevertheless, the condition had a severe clinical and quality-of-life impact, as reflected by widespread intensely pruritic lesions, incessant crying, marked sleep disturbance, and a substantial burden on both the infant and his family. After 40 days of emollient therapy, skin-barrier care, and topical corticosteroids during inflammatory flares, the SCORAD score decreased to 18, corresponding to mild disease and representing a 60% reduction from baseline. The eczematous morphology, marked pruritus, familial atopic background, and therapeutic response supported early-onset atopic dermatitis rather than a typical CMV-associated cutaneous eruption. Nevertheless, the coexistence of CMV seropositivity and atopic dermatitis does not establish a causal relationship between them.

Unusually early eczematous dermatitis associated with impaired growth or systemic manifestations should prompt consideration of alternative diagnoses, including monogenic atopic disorders, genetic skin-barrier defects, and inborn errors of immunity [10]. Netherton syndrome remained an important differential diagnosis because it may present with congenital erythroderma, failure to thrive, and atopic manifestations. Characteristic ichthyosis linearis circumflexa and clinically apparent hair abnormalities were not identified; however, hair-shaft microscopy for trichorrhexis invaginata and SPINK5 genetic testing were not performed, precluding its formal exclusion.

Infantile seborrhoeic dermatitis was considered less likely because of the extensive pruritic eczematous involvement and response to topical anti-inflammatory treatment. Scabies was clinically unlikely in the absence of characteristic burrows, typical acral involvement, or affected household contacts, although skin scraping was not performed. Zinc or biotin deficiency was also considered less likely because there was no alopecia, persistent diarrhea, or typical periorificial or acral dermatitis; however, serum zinc and biotin levels were not measured. Skin biopsy and absolute eosinophil count were also unavailable. Normal age-adjusted immunoglobulin levels, total serum IgE of 10 kU/L, serum protein electrophoresis, and lymphocyte subsets, together with the absence of recurrent infections, chronic diarrhea, or clinically apparent food allergy following age-appropriate complementary feeding, were reassuring but did not exclude all forms of immune dysregulation.

The multisystem manifestations observed in this infant should be considered within the broad phenotypic spectrum reported in children with symptomatic cCMV [11]. However, because several of these abnormalities are nonspecific, alternative or concomitant diagnoses must be considered rather than attributing the entire phenotype to CMV.

Arthrogryposis represents a separate and important component of this phenotype. It is a heterogeneous clinical finding resulting from reduced fetal movement and may be associated with neurological, neuromuscular, genetic, maternal, or mechanical factors [12,13]. The combination of oligohydramnios, congenital multiple joint contractures, impaired growth, microcephaly, diffusely gracile and under-mineralized long bones, and a serum creatine kinase level of 329 U/L raises the possibility of fetal akinesia deformation sequence or an underlying congenital neuromuscular or syndromic disorder. Oligohydramnios may also have contributed to or aggravated fetal constraint and the resulting deformation sequence.

Although neurological examination and electromyography were normal at six months, these findings do not exclude congenital myopathy, congenital muscular dystrophy, motor-neuron disease, a neuromuscular-junction disorder, or another genetically determined condition during early infancy. Comprehensive molecular evaluation, including an arthrogryposis or neuromuscular gene panel and/or exome sequencing, was not performed. The underlying cause of the congenital contractures therefore remains unresolved.

Congenital CMV is not an established direct cause of arthrogryposis except potentially through severe fetal central nervous system injury and consequent reduction in fetal movement. In this infant, MRI showed no major structural brain abnormality, and neurological examination was normal. The arthrogryposis should therefore not be attributed directly to CMV. The fetal-akinesia phenotype and the unconfirmed congenital CMV hypothesis may represent coexisting but etiologically distinct diagnostic considerations.

The skeletal survey demonstrated diffusely reduced bone mineralization and gracile long bones, whereas vitamin D and calcium-phosphate parameters were within the age-appropriate reference ranges. These radiographic findings were nonspecific and may reflect prematurity, reduced fetal and postnatal mobility, nutritional history, or an underlying systemic, neuromuscular, or genetic disorder. They should not be considered evidence of CMV-related skeletal disease.

CMV-associated sensorineural hearing loss may be present at birth or develop later and may follow a progressive or fluctuating course [14-16]. However, because congenital CMV was not confirmed in this infant, the observed auditory abnormalities cannot be attributed definitively to CMV. Repeat auditory brainstem response testing and long-term audiological monitoring remain warranted. Continued ophthalmological, neurological, neurodevelopmental, dermatological, nutritional, and growth surveillance is also necessary.

This case has several important limitations. Congenital CMV infection could not be confirmed because virological testing was not performed within the first 21 days of life, no archived neonatal dried blood spot was available, maternal serology lacked IgG avidity testing and documented seroconversion, and postnatal acquisition through breastfeeding could not be excluded. Subsequent urine, saliva, or blood CMV PCR and repeat IgG testing after 12 months were not performed. The dermatological assessment did not include hair-shaft microscopy, SPINK5 testing, skin scraping or biopsy, serum zinc or biotin measurements, or an absolute eosinophil count. Comprehensive genetic and neuromuscular testing was also unavailable despite the fetal-akinesia phenotype. Finally, the MRI and neurophysiological findings were nonspecific, while the six-month follow-up and single-case design preclude conclusions regarding long-term outcomes or causal relationships among CMV seropositivity, atopic dermatitis, arthrogryposis, and the other multisystem manifestations.

## Conclusions

This case describes an infant first evaluated at six months of age with a complex phenotype comprising congenital arthrogryposis and a rash present at birth, neonatal jaundice and mild thrombocytopenia, microcephaly, impaired growth, reduced bone mineralization, neurophysiological abnormalities, and moderate early-onset atopic dermatitis with severe clinical and quality-of-life impairment. Maternal and infant CMV seropositivity, together with manifestations documented from birth, raised reasonable clinical suspicion of congenital CMV infection. Nevertheless, congenital infection remained unconfirmed because direct virological testing was not performed within the first 21 days of life, no archived neonatal dried blood spot was available, and postnatal acquisition through breastfeeding could not be excluded.

This case underscores the importance of timely targeted CMV testing in neonates with otherwise unexplained multisystem abnormalities. Confirmation within the first 21 days enables appropriate disease staging, consideration of antiviral treatment when indicated, and structured long-term audiological, ophthalmological, neurological, and developmental surveillance. The eczematous phenotype was most consistent with early-onset atopic dermatitis and should not be attributed directly to CMV. Likewise, the arthrogryposis and broader fetal-akinesia phenotype may reflect an independent congenital neuromuscular or syndromic disorder, particularly in the absence of severe structural central nervous system involvement and comprehensive genetic testing.
